# Uncovering the Role of the *KANADI* Transcription Factor *ZmKAN1* in Enhancing Drought Tolerance in Maize

**DOI:** 10.3390/plants15010002

**Published:** 2025-12-19

**Authors:** Sidi Xie, Ran Tian

**Affiliations:** 1 Dazhou Key Laboratory of Agricultural Resources Development and Ecological Conservation in Daba Mountain, Sichuan University of Arts and Science, Dazhou 635000, China; 2 School of Tourism and Culture Industry, Chengdu University, Chengdu 610106, China

**Keywords:** maize, drought tolerance, KANADI, mutant, transcriptome

## Abstract

Drought stress causes substantial yield losses in maize, posing a serious threat to food security. Leaf adaxial-abaxial polarity development is closely associated with drought tolerance. *KANADI* (*KAN*) genes play a pivotal role in leaf polarity establishment and are likely involved in regulating drought tolerance in maize. In this study, we identified 11 *ZmKAN* genes through sequence similarity analysis and functionally characterized one of them, *ZmKAN1*, in the context of drought response. The *kan1-1* mutant exhibited enhanced drought tolerance compared to the wild-type B73. Transcriptome analysis revealed that differentially expressed genes in the mutant before and after drought stress were significantly enriched in pathways associated with drought tolerance, including “response to heat”, “secondary metabolite biosynthetic process”, and “plant hormone signal transduction”, suggesting that *ZmKAN1* may modulate maize drought tolerance by regulating key processes such as heat response and plant hormone signaling. Furthermore, the differentially expressed genes in the wild type before and after drought stress were enriched in pathways such as “structural constituent of ribosome”, “mitochondrial respiratory chain complex I”, and “ribosome”, suggesting that drought stress may impair ribosomal and mitochondrial functions more severely in the wild type, along with other cellular organelles. In contrast, mutants exhibited relatively stable ribosomal and mitochondrial activities, enabling them to maintain higher survival rates and enhanced drought tolerance under drought conditions. Our findings provide important insights into the molecular mechanisms underlying drought tolerance in maize and offer valuable genetic resources for breeding drought-resistant maize cultivars.

## 1. Introduction

Maize (*Zea mays*) is one of the most important cereal crops worldwide [1,2]. Despite its widespread cultivation, maize productivity is persistently limited by abiotic stresses, with drought being one of the most devastating factors [3]. Water deficit adversely affects various physiological, biochemical, and developmental processes in maize, leading to significant yield losses and jeopardizing food security in many regions. Consequently, the development and deployment of drought-tolerant and high-yielding maize varieties constitute a major scientific challenge in ensuring food security. Breeding novel maize varieties with enhanced drought tolerance and yield potential is therefore crucial for achieving stable and increased grain production.

Leaf polar development, which governs the asymmetric patterning of the adaxial and abaxial sides of the leaf, is critically linked to plant drought tolerance. Under drought stress, polarity-related genes orchestrate dynamic transcriptional reprogramming that promotes structural remodeling of leaves—such as altered stomatal distribution, thickened cuticles, and enhanced vascular organization—thereby conferring improved drought resistance [4,5,6]. In recent years, several key regulators of leaf polarity have been molecularly cloned and functionally characterized, some of which have already been applied in genetic improvement of crops [7]. Consequently, the discovery and functional elucidation of additional leaf polarity genes offer substantial potential for guiding the breeding new maize varieties with enhanced drought tolerance and superior yield performance.

The establishment of leaf adaxial-abaxial polarity is a fundamental process in plant morphogenesis, governed by a complex genetic network. Key regulators implicated in this process include several conserved transcription factors and small RNA pathways. Among them, the *CLASS III HOMEODOMAIN-LEUCINE ZIPPER* (*HD-ZIPIII*) family genes promote adaxial identity [8], while the *KAN1* family specifies abaxial fate [9]. Similarly, *YABBY* (*YAB*) transcription factors act as critical determinants of abaxial development [10]. The *ASYMMETRIC LEAVES1* (*AS1*) and *AS2* represses abaxial-promoting genes and reinforces adaxial identity [11]. Additionally, *AUXIN RESPONSE FACTORS 3* and *4* (*ARF3/4*) contribute to abaxial patterning [12]. Small RNA pathways also play essential roles: microRNAs (miRNAs) such as miR165/166 post-transcriptionally inhibit *HD-ZIPIII* transcripts to promote abaxialization [13], and trans-acting small interfering RNAs (ta-siRNAs) facilitate the formation of adaxial tissue by repressing *ARF3/4* expression [14]. A summarized model suggests that *HD-ZIPIII*, *AS1*/*AS2*, and ta-siRNA pathways synergistically promote adaxial fate, whereas miR165/166, *KAN1*, *YAB*, and *ARF3*/*4* collectively specify abaxial identity, forming a reciprocal regulatory circuit that ensures robust polarity establishment [15].

*KANADI*, a member of the GARP (GOLDEN2, *Arabidopsis* Response Regulator (ARR) and Phosphorus Stress Response1 (PSR1)) family of transcription factors, is predominantly expressed in the abaxial side of leaf primordia and functions to repress the transcription of *HD-ZIPIII* genes [9,16,17]. In loss-of-function *kanadi* mutants, leaves exhibit adaxialized phenotypes, accompanied by ectopic upregulation of *HD-ZIPIII* expression in the abaxial tissues. In rice, loss-of-function mutations in the *KANADI* family gene *ROLLED LEAF9* (*RL9*)/*SHALLOT-LIKE1* (*SLL1*) lead to the replacement of sclerenchyma cells on the abaxial side of leaf vascular bundles with mesophyll cells. This results in disrupted adaxial-abaxial patterning and inwardly rolled leaves [18,19]. Conversely, overexpression of *KANADI* results in abaxialized characteristics on the adaxial side of leaves, demonstrating its central role in establishing abaxial-adaxial polarity [9,20,21].

Plants have been extensively studied for their responses to drought at morphological, physiological, biochemical, and molecular levels [22]. To adapt to arid environments, plants have evolved multiple strategies, including leaf rolling to reduce transpirational water loss, thereby enhancing drought tolerance [23]. Plants can also minimize water loss and improve water uptake by adjusting stomatal density and aperture, modifying canopy size and root architecture, among other adaptations [22,24]. At the cellular level, plants undergo a series of adjustments to protect themselves from damage, such as producing hydrophilic proteins, detoxifying enzymes, and osmoprotectants. These protective mechanisms are typically regulated transcriptionally and metabolically. The accumulation of endogenous abscisic acid (ABA) plays a pivotal role in drought responses. In the presence of ABA, SNF1-RELATED PROTEIN KINASE 2s (SnRK2s) are activated and subsequently phosphorylate and activate downstream targets, including S-type anion channels, NADPH oxidases, and ABA-responsive transcription factors, all of which contribute to the regulation of growth and adaptation under drought conditions [25,26,27,28]. Numerous studies have elucidated the roles of transcription factors from various families in drought signal transduction in maize [29,30]. For instance, NAC (NAM, ATAF, and CUC) transcription factors play crucial roles in plant drought tolerance. Overexpression of *ZmNAC* family genes, such as *ZmNAC48*, *ZmNAC49*, and *ZmNAC111*, has been shown to significantly enhance drought tolerance in maize [31,32,33]. The identification of these genes is of great significance for enhancing drought tolerance in maize. However, the current understanding of drought tolerance-related genes in maize remains incomplete, and the exploration of additional genes and regulatory mechanisms is crucial for developing drought-resistant maize varieties. *KANADI* plays an important role in leaf polarity development [9,20,21], and leaf polarity development has been closely linked to plant drought tolerance [4,5,6]. The *KANADI* gene potentially participate in regulating drought tolerance in maize.

In this study, we successfully identified 11 *KANADI* family genes in maize by performing sequence similarity-based searches using known rice components. Notably, *Zm00001d032249* and *Zm00001d050350* were found to be homologs of the rice *KANADI* gene *RL9*/*SLL1*, which is known to cause abnormalities in leaf adaxial-abaxial polarity development, suggesting their potential role in regulating drought tolerance in maize. Based on expression profile analysis, *Zm00001d032249*, which exhibited higher expression levels in leaves, was selected for functional characterization in drought stress response. The mutant of this gene showed significantly enhanced drought tolerance. Subsequent transcriptome analysis identified the regulatory pathways and downstream genes through which *Zm00001d032249* influences drought tolerance, revealing its critical role in the regulation of drought stress in maize. This study not only provides important insights into the molecular mechanisms underlying drought tolerance in maize but also offers valuable gene resources and germplasm materials for breeding new drought-tolerant maize varieties.

## 2. Results

### 2.1. Identification of Maize KANADI Family Genes

**Figure 1:**
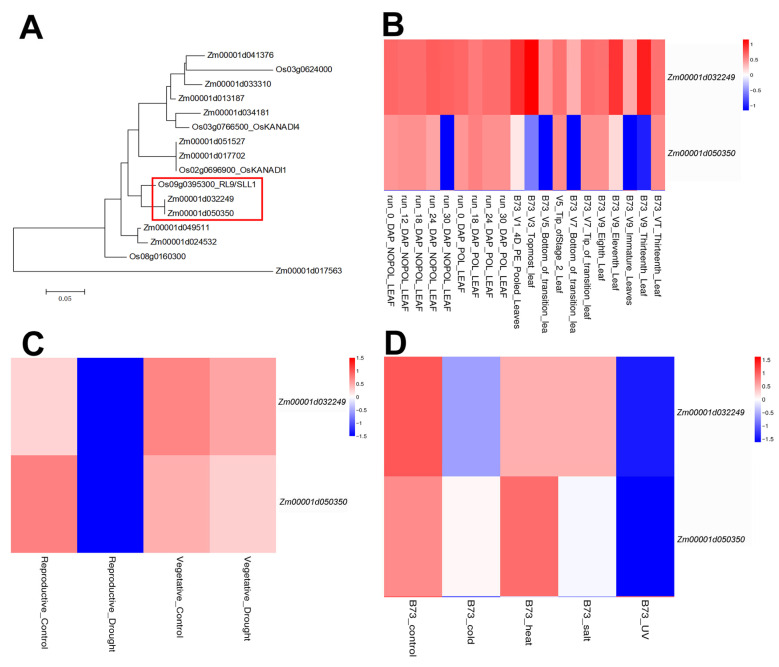
Identification of *KANADI* family genes in maize. (**A**) Phylogenetic tree of maize and rice *KANADI* family genes. Evolutionary distances were estimated with a neighbor-joining algorithm. The scale bar indicates the average number of amino acid substitutions per site. The red boxes highlight two maize *KANADI* genes that exhibit a closer phylogenetic relationship to the rice *RL9*/*SLL1* gene. (**B**) The expression patterns of *Zm00001d032249* and *Zm00001d050350* in leaves. (**C**) The expression patterns of *Zm00001d032249* and *Zm00001d050350* under drought stress. (**D**) The expression patterns of *Zm00001d032249* and *Zm00001d050350* under stresses including cold, heat, salt, and ultraviolet radiation.

A total of 11 maize *KANADI* family genes were identified by searching the maize genome annotation database using BLASTp analysis with the known protein sequences of *KANADI* family genes from rice as queries. Among these, *Zm00001d032249* and *Zm00001d050350* exhibited closer phylogenetic relationships to the rice *RL9*/*SLL1* gene, mutations in which are known to cause aberrant leaf adaxial-abaxial polarity development (Figure 1A). The amino acid sequence similarity between Zm00001d032249 and Zm00001d050350 is 75.37% (Figure S1). This suggests that these two genes may also play significant roles in regulating adaxial-abaxial polarity in maize leaves. We further analyzed the expression patterns of these two genes in leaves using transcriptomics data from qTeller (https://qteller.maizegdb.org/; accessed on 3 August 2025). *Zm00001d032249* showed relatively higher expression levels in leaves (Figure 1B, Table S1). We also conducted an analysis of the expression of these two genes under different stresses. The expression levels of both genes underwent down-regulation under drought stress during the reproductive stage, which suggests that both genes may participate in drought regulation (Figure 1C, Table S1). In addition, under stresses such as cold, heat, salt, and ultraviolet radiation, the gene expression patterns experience substantial changes, which might also have a certain regulatory effect on these stresses (Figure 1D, Table S1). Furthermore, the promoters of *Zm00001d032249* and *Zm00001d050350* were analyzed via the Plantpan4.0 database (http://plantpan.itps.ncku.edu.tw/plantpan4/index.html; accessed on 8 August 2025). The ARR-B family transcription factors, B3 family transcription factors, CPP family transcription factors, and TCP family transcription factors were exclusively identified in the *Zm00001d032249* promoter, which might be associated with the differential expression of the two genes (Tables S2 and S3). In this study, we selected *Zm00001d032249*, a gene that exhibits a relatively high expression level in leaves, to investigate its drought resistance regulatory function and potential mechanism. This gene was designated as *ZmKAN1*.

### 2.2. The ZmKAN1 Mutant Exhibits Enhanced Drought Tolerance

**Figure 2:**
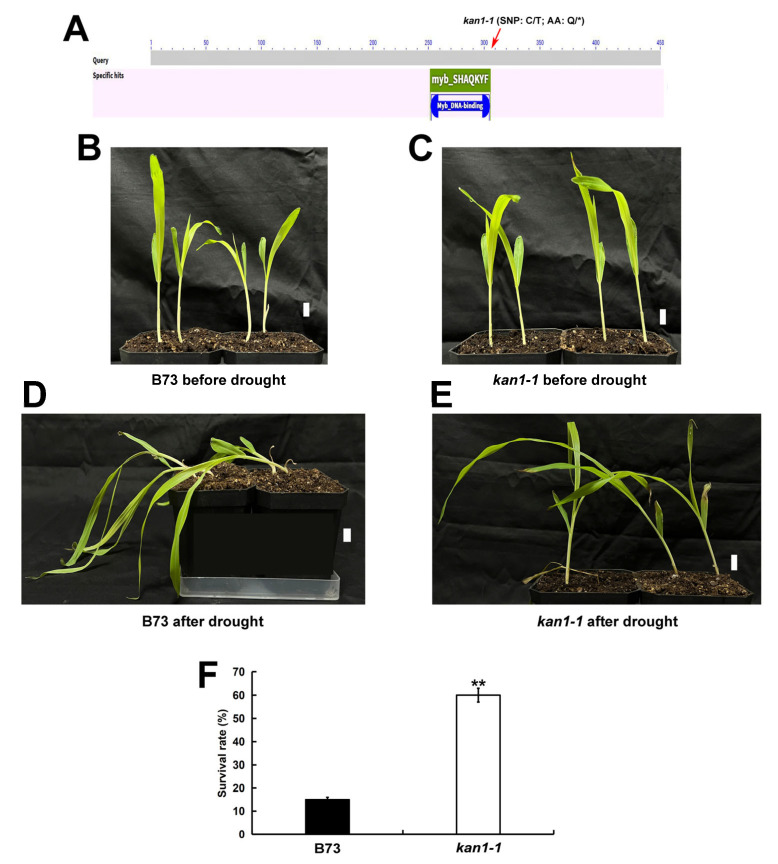
The *ZmKAN1* mutant exhibits enhanced drought tolerance compared to the wild type. (**A**) Schematic diagram of *ZmKAN1* with indicated mutation sites and protein conserved domains. SNP, single-nucleotide polymorphism; AA, amino acids; C, cytosine; T, thymine; Q, glutamine; *, stop gained. (**B**) Wild-type B73 seedlings before drought stress. (**C**) Mutant *kan1-1* seedlings before drought stress. (**D**) Following drought stress, all wild-type B73 seedlings exhibited severe wilting and lodging. (**E**) The survival rate of *kan1-1* mutant seedlings was significantly higher than that of wild-type seedlings following drought stress treatment. Scale bar, 1 cm. (**F**) Survival rate of B73 and *kan1-1* after drought tolerance. The data are means ± SD (*n* = 3). ** significant at *p* < 0.01 by Student’s *t* test. The culture conditions were set at 25 °C, with a photoperiod of 16 h light and 8 h dark. Watering was halted when the seedlings reached 14 days of age, and the survival rate of the seedlings was assessed when the soil water content was around 2%.

A mutant of *ZmKAN1*, designated *kan1-1*, was further characterized. This mutant was generated by ethylmethane sulfonate (EMS) mutagenesis of the maize inbred line B73. The mutation corresponds to a C-to-T substitution in exon 2 of *ZmKAN1*, resulting in the conversion of a glutamine residue to a premature termination codon. Conserved domain analysis indicated that this mutation is located in the tail region of the MYB DNA-binding domain. Notably, the mutant protein retains the intact DNA-binding domain, suggesting that partial function of the gene may be preserved (Figure 2A). Subsequent analysis compared the growth of wild-type B73 and *kan1-1* mutant seedlings under drought stress (Figure 2B–E). Following drought treatment, wild-type B73 seedlings exhibited pronounced lodging and wilting (Figure 2D), the survival rate is 15% (Figure 2F). In contrast, the survival rate of *kan1-1* mutant seedlings was 60%, significantly higher than that of the wild-type B73 (Figure 2E,F). These results demonstrate that the *kan1-1* mutant possesses enhanced drought tolerance, indicating that *ZmKAN1* functions in regulating drought tolerance in maize.

### 2.3. Analysis of ZmKAN1 Expression Levels

**Figure 3:**
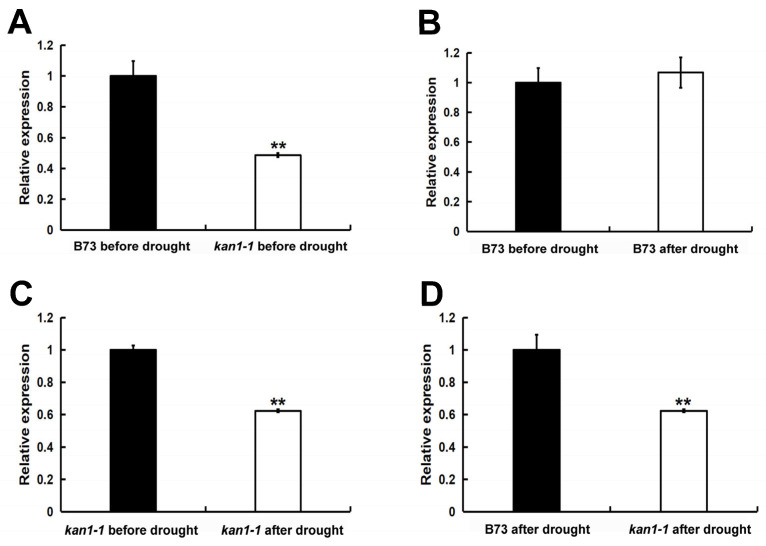
The expression levels of *ZmKAN1*. (**A**) The expression levels of *ZmKAN1* in B73 and *kan1-1* before drought stress. (**B**) The expression levels of *ZmKAN1* in B73 under normal conditions and after drought stress. (**C**) The expression levels of *ZmKAN1* in *kan1-1* under normal conditions and after drought stress. (**D**) The expression levels of *ZmKAN1* in B73 and *kan1-1* after drought stress. The data are means ± SD (*n* = 3). ** significant at *p* < 0.01 by Student’s *t* test.

Given that the *kan1-1* mutant might retain partial *ZmKAN1* function, we analyzed *ZmKAN1* expression in wild-type B73 and the *kan1-1* mutant under both well-watered conditions and after a 24-h drought treatment. Under well-watered conditions, *ZmKAN1* expression in the mutant was significantly lower than in the wild type, indicating that the mutation compromises its expression (Figure 3A). While drought stress did not alter *ZmKAN1* expression levels in the wild type, a highly significant reduction was observed in the mutant following the stress treatment (Figure 3B,C). Specifically, compared to its pre-stress level, *ZmKAN1* expression in the mutant was dramatically down-regulated after drought stress (Figure 3C). Furthermore, post-drought stress, the mutant exhibited significantly lower *ZmKAN1* expression compared to the stressed wild-type plants (Figure 3D). Collectively, these results demonstrate that the mutation renders *ZmKAN1* expression responsive to drought stress.

### 2.4. Transcriptome Profiling

**Figure 4:**
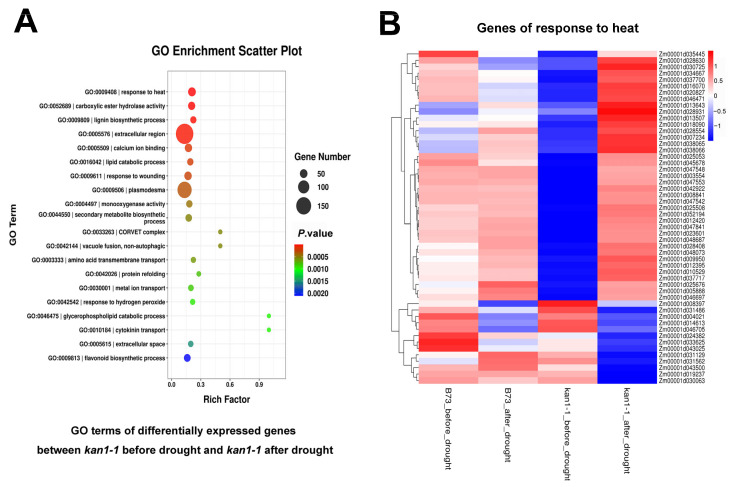
GO enrichment analysis of differentially expressed genes in *kan1-1* before and after drought treatment, along with a heat map displaying the expression patterns of genes in the GO term “response to heat”. (**A**) GO terms of differentially expressed genes in *kan1-1* before and after drought treatment. (**B**) Heat map depicting the expression levels of genes in the GO term “response to heat”. These genes exhibited highly significant differential expression in *kan1-1* before and after drought stress.

**Figure 5:**
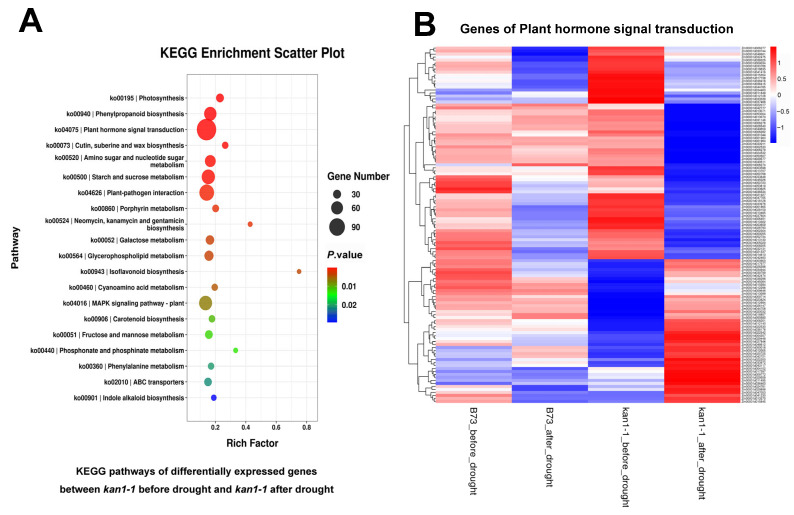
KEGG enrichment analysis of differentially expressed genes in *kan1-1* before and after drought treatment, along with a heat map displaying the expression patterns of genes in the KEGG pathway “plant hormone signal transduction”. (**A**) KEGG pathways of differentially expressed genes in *kan1-1* before and after drought treatment. (**B**) Heat map depicting the expression levels of genes in the KEGG pathway “plant hormone signal transduction”. The majority of these genes exhibited significant differential expression in *kan1-1* before and after drought stress.

Transcriptome analysis was performed on wild-type B73 and mutant *kan1-1* seedlings under both well-watered conditions and after 24 h of drought stress. Differentially expressed genes (DEGs) were identified in the mutant under both conditions, followed by Gene Ontology (GO) and Kyoto Encyclopedia of Genes and Genomes (KEGG) enrichment analyses of these DEGs. GO enrichment analysis revealed that significantly enriched terms included “response to heat”, “carboxylic ester hydrolase activity”, “monooxygenase activity” and “secondary metabolite biosynthetic process”, which may be associated with drought tolerance (Figure 4A). We further examined the expression patterns of genes within the “response to heat” pathway, as heat-responsive genes have been reported to play important roles in drought stress responses. These genes exhibited highly significant differential expression in the mutant before and after drought stress, whereas no significant differences were observed in the wild-type under either condition. This finding suggests a potential mechanism contributing to the enhanced drought tolerance observed in the mutant (Figure 4B). KEGG pathway analysis indicated significant enrichment in pathways such as “photosynthesis”, “phenylpropanoid biosynthesis”, “plant hormone signal transduction”, and “cutin, suberine and wax biosynthesis” (Figure 5A). Further analysis focused on genes within the “plant hormone signal transduction” pathway, given the crucial role of phytohormones, such as ABA, in regulating drought tolerance. Similarly, the majority of these genes showed highly significant differential expression in the mutant before and after drought stress, but not in the wild-type, which may represent another factor underlying the superior drought tolerance of the mutant (Figure 5B).

**Figure 6:**
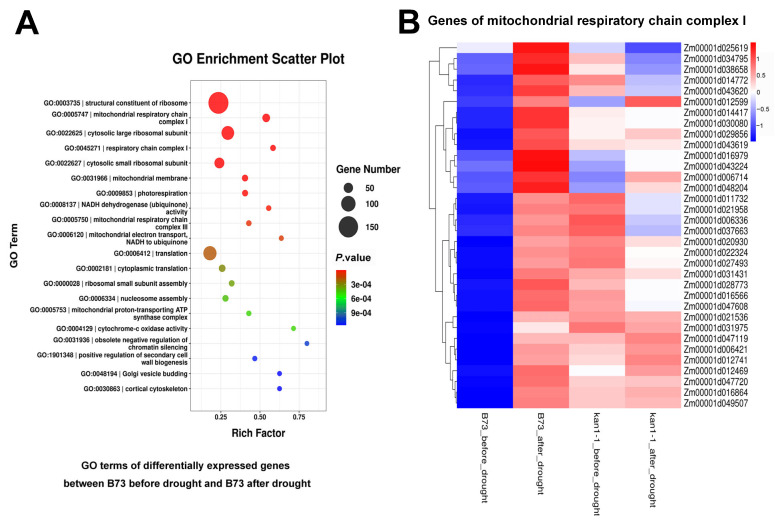
GO enrichment analysis of differentially expressed genes in B73 before and after drought treatment, along with a heat map displaying the expression patterns of genes in the GO term “mitochondrial respiratory chain complex I”. (**A**) GO terms of differentially expressed genes in B73 before and after drought treatment. (**B**) Heat map depicting the expression levels of genes in the GO term “mitochondrial respiratory chain complex I”. The expression levels of these genes were significantly upregulated in B73 following drought stress.

**Figure 7:**
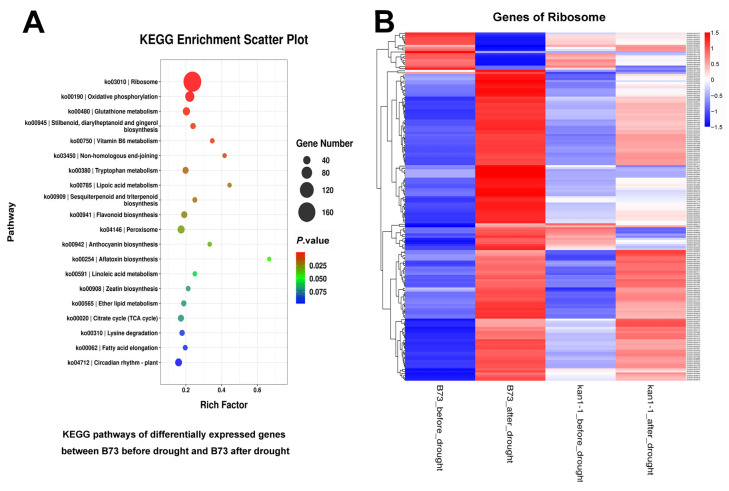
KEGG enrichment analysis of differentially expressed genes in B73 before and after drought treatment, along with a heat map displaying the expression patterns of genes in the KEGG pathway “Ribosome”. (**A**) KEGG pathways of differentially expressed genes in B73 before and after drought treatment. (**B**) Heat map depicting the expression levels of genes in the KEGG pathway “Ribosome”. These genes exhibited significant differential expression in B73 before and after drought stress.

Additionally, DEGs in the wild-type under both conditions were identified, followed by GO and KEGG enrichment analyses. Significantly enriched GO terms included “structural constituent of ribosome”, “mitochondrial respiratory chain complex I”, “cytosolic large ribosomal subunit” and “respiratory chain complex I”, suggesting that drought stress may cause more severe damage to organelles such as ribosomes and mitochondria in the wild-type, leading to its reduced survival rate after drought stress (Figure 6A). Further analysis of genes within the “mitochondrial respiratory chain complex I” pathway revealed that they were all significantly up-regulated in the wild-type following drought stress, whereas no notable changes were observed in the mutant under either pre- or post-drought conditions (Figure 6B). This may enable the mutant to maintain relatively stable ribosomal and mitochondrial functions under drought stress, thereby contributing to its higher survival rate. Significantly enriched KEGG pathways included “ribosome” and “oxidative phosphorylation” (Figure 7A). Further examination of genes in the “ribosome” pathway showed highly significant differential expression in the wild-type under pre- and post-drought conditions, while most genes in the mutant exhibited no significant changes (Figure 7B). These findings suggest that ribosomal function in the wild-type is more severely impaired under drought stress, which may further explain its lower survival rate. In addition, we also verified the expression levels of *HEAT SHOCK TRANSCRIPTION FACTOR1* (*HSF1*), *HEAT SHOCK PROTEIN7* (*HSP7*), *SnRK2.6* and *SnRK2.7* by qRT-PCR, and the results were consistent with the transcriptome data (Figure S2).

## 3. Discussion

### 3.1. ZmKAN1 Modulates Drought Tolerance in Maize at the Seedling Stage

Following drought stress, the survival rate of the *kan1-1* mutant seedlings was significantly higher than that of the wild-type B73 (Figure 2), demonstrating that *ZmKAN1* functions in regulating drought tolerance during the maize seedling stage. Previous studies have shown that the *Arabidopsis KAN1* regulates the expression of multiple ABA signaling-related genes, including members of the ABA receptor family and SnRK2/SnRK3 protein kinase families [34]. The phytohormone ABA and SnRK2 kinases, among others, play crucial roles in plant drought tolerance [25,26,27,28], suggesting that *KAN1* may influence drought tolerance by modulating ABA signaling and associated genes. Furthermore, *Populus trichocarpa KAN1* is specifically highly expressed in roots and influences lateral root development [35]. The expansion capacity of lateral roots is a key trait for plant drought tolerance, as it enhances water uptake efficiency [36]. These findings collectively support the notion that plant *KAN1* genes may affect drought tolerance by regulating key drought-responsive genes or traits. Additionally, the *kan1-1* mutant retains an intact DNA-binding domain in the *ZmKAN1* gene, potentially preserving partial function (Figure 2A). Further analysis revealed that *ZmKAN1* expression was significantly lower in the mutant after drought stress compared to pre-stress levels (Figure 3C), whereas its expression remained unchanged in the wild-type under both conditions (Figure 3B). We hypothesize that *ZmKAN1*, as a transcription factor, may bind to its own promoter to autoregulate its expression. Analysis of the *ZmKAN1* promoter using the Plantpan4.0 database (http://plantpan.itps.ncku.edu.tw/plantpan4/index.html; accessed on 30 August 2025) identified 18 potential GARP family transcription factor binding sites, including one for *ZmKAN1* itself (Table S4). This indicates that *ZmKAN1* may autoregulate its expression, and suppression of its expression may be associated with enhanced drought tolerance. These findings provide novel insights and a potential target for developing new drought-tolerant germplasm based on *ZmKAN1*.

### 3.2. ZmKAN1 Potentiates Drought Tolerance Likely via Modulating Heat Response and Plant Hormone Pathways

GO and KEGG enrichment analyses revealed that the DEGs in the mutant, before and after drought stress, were significantly enriched in pathways closely associated with drought response, such as “response to heat” and “plant hormone signal transduction” (Figure 4 and Figure 5). Heat-responsive genes have been documented to play crucial roles in coping with drought stress. For instance, *HSF1* (*Zm00001d005888*), *ASCORBATE PEROXIDASE2* (*Zm00001d007234*), *ANNEXIN2* (*Zm00001d018090*), and *HSP7* (*Zm00001d012395*) have all been reported to regulate drought tolerance in maize [37,38,39,40]. These four genes exhibited highly significant upregulation in the mutant after drought stress, whereas no significant differences were observed in the wild-type between well-watered and drought conditions. This specific upregulation in the mutant likely contributes, at least partially, to the enhanced drought tolerance of *kan1-1*. Genes enriched in the “plant hormone signal transduction” pathway included key players in ABA signaling, such as the protein kinases *SnRK2.6* (*Zm00001d050723*), *SnRK2.7* (*Zm00001d003659*), *SnRK2.9* (*Zm00001d033339*), and *SnRK2.11* (*Zm00001d038326*), which are also critically involved in the regulation of maize drought tolerance [28,41]. These differentially expressed genes might serve as the direct regulatory genes of ZmKAN1. For instance, the promoter of *HEAT SHOCK PROTEIN7* was analyzed using the Plantpan4.0 database (http://plantpan.itps.ncku.edu.tw/plantpan4/index.html; accessed on 9 December 2025). This promoter contains the binding sites of multiple GARP family transcription factors, and ZmKAN1 is a potential upstream transcription factor of *HEAT SHOCK PROTEIN7* (Table S5).

Furthermore, DEGs identified in the wild-type, before and after drought stress, were primarily enriched in pathways related to the ribosome, mitochondrion, and associated functions. The expression of these related genes showed highly significant differences between the two conditions in the wild-type. However, most of these genes displayed no significant expression changes in the mutant before versus after stress (Figure 6 and Figure 7). Previous studies have established that mitochondria influence plant drought tolerance by modulating cellular redox homeostasis, energy metabolism, and antioxidant defense [42,43,44]. Similarly, maintaining the homeostasis of ribosomal proteins or regulating the expression of ribosomal proteins can promote drought tolerance [45,46,47]. Our findings suggest that cellular components like ribosomes and mitochondria may suffer greater disruption in the wild-type under drought stress. Consequently, this heightened susceptibility likely leads to the lower survival rate observed in the wild-type following drought stress. The relative stability of ribosome and mitochondrial functions in the *kan1-1* mutant may thus represent another fundamental reason for its superior drought tolerance.

## 4. Materials and Methods

### 4.1. Plant Materials

The materials used in this study included maize inbred line B73 (stored within our laboratory) and an EMS-mutagenized stop-gained mutant (EMS4-0d34ed, *kan1-1*) which were obtained from the Maize EMS-induced Mutant Database (MEMD; http://maizeems.qlnu.edu.cn/; accessed on 5 August 2025) [48].

### 4.2. Identification of KANADI Family Genes in Maize

The maize genome dataset was obtained from maizeGDB (Maize Genetics and Genomics Database, https://www.maizegdb.org/; accessed on 3 August 2025). A BLASTp (version 2.11.0+) search was performed using known KANADI family protein sequences from rice to identify putative KANADI family genes in maize.

### 4.3. Phylogenetic Analysis and Gene Expression Patterns Analysis

Protein sequences of the *KANADI* family genes in maize were downloaded from maizeGDB, while those from *Arabidopsis* were obtained from the National Center for Biotechnology Information (NCBI; https://www.ncbi.nlm.nih.gov/; accessed on 3 August 2025). Multiple sequence alignment was performed using MUSCLE implemented in MEGA software (version 5.1.1). A phylogenetic tree was constructed by the neighbor-joining method, and the evolutionary distances were evaluated with bootstrap testing based on 1000 replicates. By leveraging the maize gene expression data from qTeller (https://qteller.maizegdb.org/; accessed on 3 August 2025), the gene expression patterns at different growth stages of leaves and under various stress treatments were analyzed.

### 4.4. Drought Tolerance Analysis of Wild-Type and Mutant Plants

Seeds of the wild-type B73 and the mutant *kan1-1* were sown in cultivation trays and grown under controlled conditions at 25 °C with a 16 h light/8 h dark photoperiod. Drought stress was initiated by withholding water for 14-day-old seedlings. After 10 days of treatment, the soil water content was measured to be approximately 2% using a soil moisture meter, and the seedling survival was assessed, and the number of surviving plants was recorded to evaluate the drought tolerance of B73 and *kan1-1*. A comparison was made among at least 15 plants of each material, and statistical analysis was carried out based on the data obtained from three independent experiments.

### 4.5. RNA Extraction and qRT-PCR Analysis

Fourteen-day-old wild-type B73 and mutant *kan1-1* seedlings were subjected to drought treatment. Leaves of B73 and *kan1-1* before drought treatment and 24 h after drought treatment were collected. Two biological replicates were collected, and all samples were immediately frozen in liquid nitrogen and stored at −80 °C. Total RNA was extracted from each sample using Trizol reagent (Invitrogen, Carlsbad, CA, USA). The expression of *ZmKAN1* was validated using qRT-PCR. The Fast Quant RT Kit (TianGen, Beijing, China) was used to synthesize the first strand cDNAs. The qRT-PCR was then conducted using the Bio-Rad iQ5 (Bio-Rad, Hercules, CA, USA) according to the SuperReal PreMix Plus (SYBR Green) instructions (TianGen, Beijing, China). All reactions were performed with three technical replicates, and the expression levels were normalized using *Glyceraldehyde-3-phosphate dehydrogenase* (*GAPDH*) as an internal reference. The qRT-PCR primers are listed in Table S6.

### 4.6. Transcriptome Analysis

Each transcriptome sequencing library was prepared using approximately 15 μg of total RNA. A total of eight transcriptome sequencing libraries were constructed using each biological replicate of the collected B73 and *kan1-1* leaf samples before and after the drought treatment. For each treatment, one healthy plant was selected, and two biological replicates were collected. Transcriptome sequencing was performed by the LC-Biotech Co., Ltd., Hangzhou, China. Data were processed and analyzed as previously described [49]. Genes with |log2(FoldChange)| > 1 and *p*-value < 0.05 were considered to have altered expression and were designated as differentially expressed genes. GO and KEGG enrichment analysis and visualization of differentially expressed genes was implemented by the clusterProfiler R package (4.10.1), which is a simple-to-use tool to analyze high-throughput data obtained from transcriptomics or proteomics. Genes with adjusted *p*-value less than 0.05 were considered significantly enriched by differential expressed genes [50]. Raw sequence data for the transcriptome in this study can be found in the China National Center for Bioinformation (https://www.cncb.ac.cn/) under accession number PRJCA049973.

## 5. Conclusions

Our study demonstrates that *ZmKAN1* regulates drought tolerance in maize at the seedling stage. The *kan1-1* mutant exhibits enhanced drought tolerance compared to the wild type. Further transcriptomic analysis identified downstream regulatory pathways of *ZmKAN1*, which may enhance drought resistance by modulating heat response, phytohormone pathways, and stabilizing organellar functions. This work provides a valuable genetic resource for optimizing drought-tolerant maize breeding.

## Data Availability

The original contributions presented in this study are included in the article/Supplementary Material. Further inquiries can be directed to the corresponding authors.

## Supplementary Materials

The following supporting information can be downloaded at: https://www.mdpi.com/article/10.3390/plants15010002/s1, Table S1: The expression data of *Zm00001d032249* and *Zm00001d050350* obtained from qTeller; Table S2: Promoter analysis of *Zm00001d032249*; Table S3: Promoter analysis of *Zm00001d050350*; Table S4: 18 potential GARP family transcription factors that may bind to the *ZmKAN1* promoter; Table S5: The potential binding sites of GARP family transcription factors within the promoter of *HEAT SHOCK PROTEIN7*; Table S6: Primers used in this study; Figure S1: Amino acid sequence alignment of Zm00001d032249 and Zm00001d050350; Figure S2: The expression levels of *HSF1*, *HSP7*, *SnRK2.6* and *SnRK2.7*.
