# Supplementary material for: Uncovering the Role of the KANADI Transcription Factor ZmKAN1 in Enhancing Drought Tolerance in Maize
Source: Plants (Basel). 2025 Dec 19;15(1):2. doi: 10.3390/plants15010002 (PMC12788164; doi:10.3390/plants15010002)
Supplement: Supplementary file 1 [file plants-15-00002-s001.zip › Supplementary Figure S1.pdf]

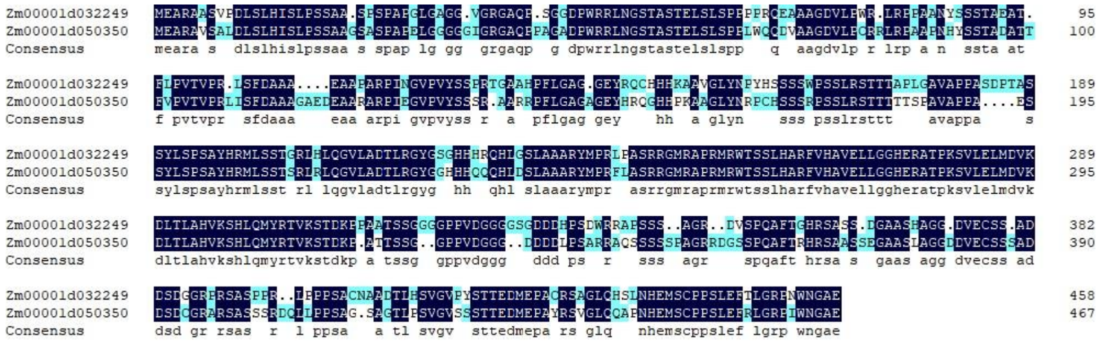


Figure S1 Amino acid sequence alignment of Zm00001d032249 and Zm00001d050350. The similarity between the two sequences is 75.37%.
