# Supplementary material for: Uncovering the Role of the KANADI Transcription Factor ZmKAN1 in Enhancing Drought Tolerance in Maize
Source: Plants (Basel). 2025 Dec 19;15(1):2. doi: 10.3390/plants15010002 (PMC12788164; doi:10.3390/plants15010002)
Supplement: Supplementary file 1 [file plants-15-00002-s001.zip › Supplementary Figure S2.pdf]

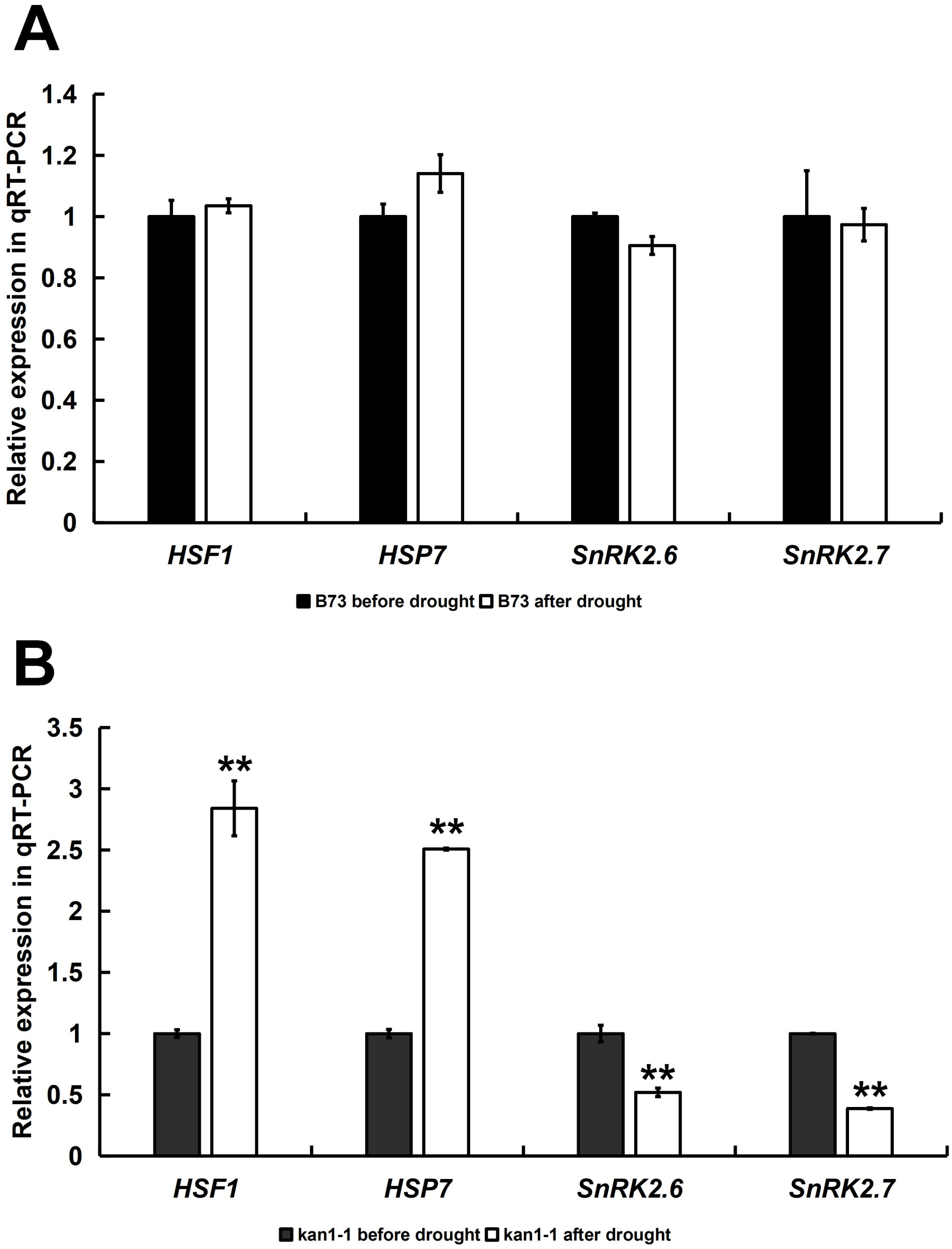

Figure S2 The expression levels of *HSF1*, *HSP7*, *SnRK2.6* and *SnRK2.7*. (**A**) The expression levels of *HSF1*, *HSP7*, *SnRK2.6* and *SnRK2.7* in B73 under normal conditions and after drought stress, there is no significant difference in gene expression levels. (**B**) The expression levels of *HSF1*, *HSP7*, *SnRK2.6* and *SnRK2.7* in *kan1-1* under normal conditions and after drought stress, there are significant differences in gene expression levels. The data are means ± SD (n=3). ** significant at $p < 0.01$ by the Student's *t* test.
